# Effects of a Mindfulness-Based Intervention on Male Portuguese Prisoners

**DOI:** 10.1177/0306624X221106333

**Published:** 2022-06-20

**Authors:** Cláudia Carmo, Vivien Iacob, Marta Brás, Jacinta Fernandes

**Affiliations:** 1 Universidade do Algarve, Campus de Gambelas, Faro, Portugal

**Keywords:** mindfulness-based intervention, prison, inmates, mental health, self-esteem

## Abstract

Mindfulness-based interventions (MBIs) in prison environments have revealed positive benefits for prisoners’ physical and psychological health. This study aimed to verify the efficacy of an MBI program in decreasing depressive symptoms, anxiety, stress, negative effects, and increasing positive affects, self-esteem, and mindfulness state and capacity in prisoners. The sample comprised 44 Portuguese male prisoners, who were divided into two groups: the mindfulness training group (*n* = 22) and the control group (*n* = 22). The mindfulness training group demonstrated increased self-esteem and mindfulness capacities. Qualitative analysis showed the usefulness of the training for inmates, not only in their daily prison life, but also post-release and the importance of breathing in coping with anxious and stressful situations. These findings suggest the benefits of MBI in prison settings and propose that these interventions may hold the potential to improve prisoners’ reintegration into society.

Incarceration can have a significant impact on mental health (Yi et al., 2017). Recent research has pointed out that inmates are more likely to have been exposed to some type of trauma (Bouw et al., 2019). They can experience significant mental health problems, especially high levels of depression, anxiety, and stress (Per et al., 2019). Approximately two out of three adult prisoners fall under some psychiatric diagnostic criteria, with a higher prevalence of major depression, trauma, stress-related disorders, anxiety disorders, and personality disorders (Fazel et al., 2016). The consequences of the high prevalence of mental illness in prisons are not only physical and psychological, but also social, with high rates of violence, suicide, and relapse (Per et al., 2019).

In response to this concern, mindfulness-based interventions (MBIs) have recently been introduced in prison environments and the results appear to be quite promising. These interventions aim to promote betterment of mental health and reduce levels of recidivism in the prison system (Samuelson et al., 2007; Simpson et al., 2019).

Mindfulness has been defined as “paying attention in a particular way; on purpose, in the present moment, nonjudgmentally” (Kabat-Zinn, 1990, 2003). The mindfulness skills or abilities could be developed by practice, mainly trained through a set of meditative exercises encouraging individuals to be aware of their internal experiences (physical feelings, thoughts, and emotions) and of the external stimulus (sounds, tastes, and smells) in a moment-by-moment base (Baer, 2003; Kabat-Zinn, 1982). Mindfulness-based programs were developed relying heavily in mindfulness meditation (Kabat-Zinn, 2003), and were introduced into therapeutic settings to provide the training and development of mindfulness skills, such as behavioral and emotional self-regulation. The awareness of the present leads the individual to have balanced and complete attention and implies a careful observation of experiences as they are, without emotional or intellectual distortions (Bishop et al., 2004).

There is growing evidence supporting the utility of MBIs in the treatment of different physical and psychological problems and, more recently, in the promotion of mental well-being (Goldberg et al., 2018). There is strong evidence that MBI reduces depression, anxiety, and stress (Strohmaier, 2020), helps in recovering from addictive behaviors and substance misuse (Li et al., 2017), improves cognitive function (Lao et al., 2016), and addresses the challenges of coping with pain (Cherkin et al., 2017). Over the past two decades, some studies have evaluated the utility of mindfulness-based programs in prison settings, supporting the potential efficacy of these interventions in inmates with a range of psychological and behavioral problems (e.g., health coping mechanism, improvements in hostility, self-esteem, and mood disturbance). Mindfulness practice has shown positive results in prison settings (Malouf et al., 2017; Shonin et al., 2013) and is an effective method for promoting self-compassion (Joss et al., 2020) and decreasing recidivism rates (Andrews & Bonta, 2010). As a therapeutic intervention, it has already been considered an integral part of the prisoners’ rehabilitation (Malouf et al., 2017; Shonin et al., 2013). Thus, mindfulness-based interventions for inmates can be important tools for improving reintegration into society and reducing criminal recidivism (Bouw et al., 2019).

Samuelson et al. (2007) investigated the effect of mindfulness-based stress reduction (MBSR) among 1.350 inmates and found a significant improvement in hostility, self-esteem, and mood disturbance. Subsequently, other studies were conducted among the prison population and showed positive results, thus emphasizing that this practice is crucial in the different aspects or dimensions of self-awareness (An et al., 2018), self-regulation (Baer, 2003), emotional regulation (Bishop et al., 2004), mood regulation (Samuelson et al., 2007), self-pity (Morley, 2018), relaxation (Fazel et al., 2016), reducing hostility (Xu et al., 2016), increasing self-esteem (Morley & Fulton, 2020), reducing substance use (Li et al., 2017), and reducing anxiety levels (Xu et al., 2016). For instance, Malouf et al. (2017) found that MBI may hold promise for reducing inmates’ risky behavior post-release, indicating that this kind of intervention may have the potential to predict post-release activities in prisoners. Simpson et al. (2019) noted significant improvements in impulsivity control, psychological well-being, resilience, and inmates’ mindfulness capacity after the program.

Reviews and meta-analyses on the effectiveness and reliability of the different MBIs in the prison context conclude that these interventions can be valid in improving prisoners’ mental health (Per et al., 2019; Yoon et al., 2017).

In a recent meta-analysis evaluating the effectiveness of MBIs in incarcerated populations, the authors concluded that MBIs promote the improvement of mental health in prisoners. The most significant results were observed in the reduction of depressive symptoms, anxiety, and stress (Per et al., 2019). On the one hand, there are demonstrations of some effectiveness of the MBIs in the prison population with small groups of prisoners all over the world, whereas on the other hand, the studies present methodological limitations and a huge heterogeneity in the type and duration of the MBI training programs. Considering that the literature is scarce regarding the implementation of MBI with defined protocols, such as mindfulness-based stress reduction (MBSR) and mindfulness-based cognitive therapy (MBCT), and that the reported results are only modestly effective, the study of the evaluation of the effectiveness of an MBI program in the adult prison population, with randomized control trials (RCTs) and the inclusion of an active control group can contribute to the knowledge about the effects of these types of programs on the mental health of the prison population.

In the current study, an MBCT program protocol was adapted for male Portuguese prisoners, piloted, and investigated for its feasibility, with three objectives—to determine the level of satisfaction of prisoners who underwent the MBI intervention; to increase participants’ levels of mindfulness; and to study the effects of the program’s practice in reducing psychologically negative symptoms (depression, anxiety, stress, and negative affects); and in improving positive aspects (self-esteem and positive affects).

## Method

### Participants

All the participants were male (male-only prison) with remaining prison sentences being no longer than 5 years, and were imprisoned for criminal behavior, mainly robbery and drug trafficking.

It was the prison director’s criterion that only inmates attending school in prison would be eligible to participate in the program. A total of 83 inmates were invited to participate, 76 (92%) agreed to participate in the program and 44 (53%) completed the study and responded to the assessment questionnaire at both time points (pre-and post-intervention).

The age of the participants ranged from 21 to 60 years old (*M* = 38.7; *SD* = 9.6); 63.6% were single, 36.4% were married or in a partnership, and 87% were Portuguese. Most of the participants had a level of education ≤9 years of schooling (89%) and only a few (11%) underwent ≥11 years of schooling.

### Procedures

The study was approved by the Portuguese Department of General Services for Reinsertion and Prison. A pre-post study design was used in this study. Pre-intervention or baseline data collection was carried out 1 week before the start of the intervention program, and post-intervention data collection was done 1 week after the program. Each participant signed an informed consent form.

The participants were assigned to two groups (the mindfulness training group, referred to as the mindfulness group and the waiting list control group, referred to as the control group). The distribution of the inmates to the groups was randomized.

Participation was voluntary and participants did not receive an award.

### Mindfulness Based Intervention

There are no published and validated specific MBI program for inmates. The MBI program was modeled after the MBCT protocol and the MBSR model. To suit the protocols to the sample were made some adjustments.

The main adaptation concerns the length of the program and the sessions. MBSR and MBCT programs had the standardized length of eight weekly, 2/2.5 hours, group sessions with one all-day retreat and daily homework practices of 40 to 60 minutes. However, MBCT and MBSR programs can be time-consuming, and question arises about their suitability for all, consequently, in recent years, different MBIs emerged concerning program length or amount of home practices and there are now different ways in which the “dose” of program is offered and/or received (Strohmaier, 2020). Due to the limitations imposed by current prison rules (such as inmate’s restrictive schedules, with very short time available/authorized for participation on additional activities), the duration of the sessions was adapted, and the 1-day retreat was excluded. Face-to-face contact with the mindfulness instructor were provided to participants in a weekly session with a duration of 90 minutes. Participants were encouraged to regularly perform the learned exercises, but all of them reported strong difficulty to find a place and a time for it in theirs currents daily life’s conditions (in prison). Trying to accomplish those limitations (shorter sessions, absence of 1-day retreat and daily practice) the program extended for 5 months, in a total of 18 sessions.

Other minor adaptation concerned the contents. The program were closest to a MBSR protocol, but a focus on cognitive approach have been retained from MBCT. The sessions were mainly experiential and aimed at developing mindfulness skills through practice, group interaction, and discussion. The format of the sessions consisted in about 60 minutes of meditative practice, and 30 minutes of feedback and psychoeducation. A range of mindfulness meditation exercises were taught (mindful yoga, body scan, sitting meditations, mindfulness walking). Every session started by mindfulness yoga practice followed by one or two other meditative practices. The final part of each session was dedicated to feedbacks. The participants were invited to describe the perceived physical sensations, thoughts, and emotions experienced during the session. The sessions were led by a licensed MBCT instructor. (For description of the sessions see Supplemental Material.)

### Psychological Measures

#### Depression Anxiety Stress Scale (DASS-21)

The Depression Anxiety Stress Scale (DASS-21; Lovibond & Lovibond, 1995) consists of 21 items divided into three subscales: depression, anxiety, and stress. Each item consists of a sentence that refers to the negative emotional symptoms experienced in the last week, as per their severity and frequency. The subscales have adequate internal consistency values in the Portuguese version (Depression α = .85, Anxiety α = .74, and Stress α = .81; Pais-Ribeiro et al., 2004) and in the present study (Depression α = .85, Anxiety α = .86, and Stress α = .85).

#### Positive and negative affect schedule (PANAS)

The positive and negative affect schedule (PANAS; Watson et al., 1988) was used to measure two dimensions of affectedness and consists of two subscales: positive and negative affect. It consists of 20 self-response items related to emotions felt during the past weeks. Both subscales showed good internal consistency in the Portuguese version (Positive Affect α = .86 and Negative Affect α = .89; Galinha & Pais-Ribeiro, 2005), and in our study (Positive Affect α = .85 and Negative Affect α = .88).

#### Rosenberg Self-Esteem Scale (RSES)

The Rosenberg Self-Esteem Scale (RSES; Rosenberg, 1965) has 10 items, with five each corresponding to negative and positive aspects. The Portuguese version of the same showed good consistency (α = .86; Santos & Maia, 2003), and the present study to (α = .84).

#### Five Facet Mindfulness Questionnaire FFMQ

The Five Facet Mindfulness Questionnaire FFMQ (Baer et al., 2006) consists of 39 items that assess the tendency (capacity) of each individual to adopt a mindfulness posture in their day-to-day lives, based on five facets or dimensions of mindfulness.

The scale showed good psychometric properties in the Portuguese version (Observe α = .78; Describe α = .88; Act consciously α = .89; Do not judge α = .86; and Do not react (α = .66; Pinto-Gouveia & Gregório, 2011) and in the present study (Observe α = .83; Describe α = .85; Do not judge α = .84; Do not react α = .84; and Act consciously α = .86).

#### Frieburg Mindfulness Inventory (FMI)

The Frieburg Mindfulness Inventory (FMI; Walach et al., 2006) measures the mindfulness state by considering it as a general construct with several interrelated facets, namely, cognitive and procedural components which include acceptance of the experience and non-judgment. The shortened version consists of 14 items. The Portuguese version (Pinto-Gouveia & Gregório, 2007) has good reliability properties. In the present study, the internal consistency was found to be α = .81.

#### Qualitative questions

Three qualitative questions were established to assess the prisoner’s perception of the intervention program. The first question ascertained how much the participants liked (or disliked) the exercises from the sessions (“Did you enjoy the exercises on the program?”), the second assessed possible perceived benefits of the program on participants’ daily life in the current prison context (“Did the practice of the exercises have an influence on your daily life in prison?”) and, the third question intended to understand whether prisoners perceived possible benefits for their daily life after release “Could practicing these exercises be helpful in your life after release?”). In all as questions it was questioned “how/what way?” and “why?”

### Data Analysis and Treatment

An intergroup analysis was performed using the non-parametric Mann-Whitney tests for two independent samples to compare the results between groups (mindfulness group and control group) at both pre-and post-intervention for all variables under analysis. The non-parametric related samples Wilcoxon matched-pairs signed-ranks test was used to assess whether there were significant changes within the groups, pre-and post-intervention. The effect sizes of the differences were estimated using Cohen’s *d*. In order to verify whether there was an association between variables at the post-intervention and for the mindfulness group, Spearman’s correlation coefficients test was performed for anxiety, depression, stress, positive and negative affect, self-esteem, and also for capacities and state of mindfulness. The Statistical Package for the Social Sciences (SPSS version 25.0 for Windows 10) was used.

The qualitative analysis was conducted based on the content of the inmates’ narratives at the end of the program in response to the open questions. The following response categories and subcategories were extracted: Experience (usefulness, difficulties, and constraints); Perceived changes in daily life (awareness, choice, serenity, and acceptance); Expected changes in life after prison (observation).

## Results

The average age for the mindfulness group was 38 years (*SD* = 11.4 years) and was found to be statistically similar to the average age of the control group (40 years; *SD* = 7.5 years). Significant differences between the two groups were found concerning literacy level, accounting for years of schooling (mindfulness group: *M* = 8.8 years, *SD* = 1.5 years; control group: *M* = 6.5, *SD* = 1.9). In the mindfulness group, 68.2% participants were single, 31.8% were married or in a partnership, and 95.5% were Portuguese. In the control group, 59.1% participants were single, 40.9% were married or in a partnership, and 90.9% were Portuguese.

### Analysis Between Groups

At the baseline (pre-intervention assessment), a few more differences between the groups were registered with regard to the psychological measures. The control group showed a significantly higher level of anxiety than the mindfulness group (*M* = 4.95, *SD* = 4.69, and *M* = 2.82, *SD* = 3.81, respectively; *U* = 152.50, *p* = .034), with a small effect size (*d* = .25). Significant intergroup differences (*U* = 130.5, *p* = .009) with a moderate effect size (*d* = −.35) were also recorded for negative affect, with that of the mindfulness group being higher than the control group (*M* = 39.86, *SD* = 10.48, and *M* = 31.82, *SD* = 11.58, respectively). In addition, self-esteem was significantly higher in the mindfulness group, with a low effect size (*U* = 158, *p* = .048, *d* = −0.31). Non-reacting mindfulness capacity was found to be significantly higher in the control group (*U* = 146, *p* = .024), with a moderate effect size (*d* = .35).

At post-intervention, no statistically significant differences between the groups were found for stress, anxiety, and depression, although the average values were lower for the mindfulness group than for the control group. Moreover, positive and negative affect differences between the groups were not significant, with the negative affect of the mindfulness group remaining higher than that of the control group (*M* = 38.45, *SD* = 10.66 and *M* = 32.50, *SD* = 13.46, respectively) similar to the pre-intervention results, although this difference was no longer significant like it was at the baseline. The self-esteem of the mindfulness group (*M* = 32.42, *SD* = 5.96) was significantly higher (*U* = 140, *p* = .016) than that of the control group (*M* = 28.55, *SD* = 4.47), with a small effect size (*d* = 0.35). No statistically significant differences between the groups at post-intervention were found for mindfulness capacities and state, although slightly higher average values were generally registered for the mindfulness group.

### Analysis Within Groups

The control group showed no statistically significant differences in any of the variables in two moments (pre-and post-intervention; Table 1).

**Table 1.** Comparative Results Between Pre- and Post-Intervention Moments for Both Groups: Mean (*M*), Standard Deviation (*SD*), and Values of the Test of Differences Moments.

Variables	Control group							Mindfulness group						
	Pre-intervention (*n* = 22)		Post-intervention (*n* = 22)		*d*	z	*p*-Value	Pre-intervention (*n* = 22)		Post-intervention (*n* = 22)		*d*	*z*	*p*-Value
	*M*	*DP*	*M*	*DP*				*M*	*DP*	*M*	*DP*			
Anxiety	4.95	4.69	4.14	4.76	0.88**	−1.543	.123	2.82	3.82	3.00	3.00	0.57	00	1
Depression	5.59	4.67	4.24	4.53	0.66**	−1.272	.203	3.50	3.82	3.63	3.37	0.76	−0.35	.723
Stress	7.14	3.80	6.32	5.21	0.91**	−1.542	.123	5.41	4.62	5.09	3.00	0.66	−0.04	.968
Positive afect	29.77	6.97	29.82	9.45	0.71**	000	1	30.86	9.61	29.50	9.53	0.65	−0.73	.465
Negative afect	31.82	11.58	32.50	13.46	0.80**	−0.526	.599	39.86	10.48	38.45	10.66	0.86	−0.93	.355
Self-esteem	29.14	4.32	28.55	4.47	0.79**	−0.911	.362	32.09	5.09	32.41	5.96	0.64	−0.51	.614
Observe	21.23	6.53	21.27	6.84	0.58**	−0.08	.936	19.82	5.60	22.50	5.60	0.53**	−2.07	.038*
Describe	26.41	5.65	26.27	6.63	0.74**	−0.24	.807	24.68	4.96	27.27	5.47	0.78**	−2.88	.004*
Act-consc	28.68	7.20	27.50	8.38	0.76**	−0.83	.407	29.63	8.07	31.27	5.31	0.60	−0.58	.565
Non-judge	28.68	6.62	27.45	7.18	0.70**	−1.24	.217	26.91	8.25	27.73	6.60	0.51	−0.26	.794
Non-react	21.63	5.53	22.05	5.10	0.58**	−0.04	.968	18.14	3.90	21.09	5.12	0.49*	−2.58	.010*
Mindful cap	128.05	17.12	126.14	19.52	0.87**	−1.01	.313	121.32	15.47	132.09	15.00	0.41	−2.24	.025*
Mindful state	39.32	5.11	38.82	6.22	0.57**	−0.65	.519	38.14	6.49	39.50	6.99	0.56	−1.04	.296

*Note.* Effect size, *d* Cohen > 0.10/29 small effect, *d* > 0.30/49 medium effect, *d* > 0.50 large effect. *M* = mean; *SD* = standart deviation; *Z* = related samples Wilcoxon matched-pairs signed-ranks test.

* *p* < .05 significant differences. **p < .01 highly significant differences

For the mindfulness group, no significant changes (moments pre-and post-intervention) were found in anxiety, depression, and stress levels. The mindfulness group’s negative and positive affect also registered no significant changes. In addition, the mindfulness group’s self-esteem increased from the baseline (*M* = 32.00, *SD* = 5.09) to post intervention (*M* = 32.41, *SD* = 5.96) but did not reach a significant level.

The mindfulness group observed significant changes (increase from pre-to post-intervention) for mindfulness capacities (*z* = −2.07, *p* = .38), with a medium effect size (*d* = 0.53), described (*z* = −2.88, *p* = .004), with a large effect size (*d* = 0.78), non-reacting (*z* = −2.58, *p* = .010), with a medium effect size (*d* = 0.49), and total mindfulness capacity (*z* = −2.24, *p* = .025) with a medium effect size (*d* = 0.42).

No significantly increased values with moderate effects sizes were obtained for consciously acting capacity, non-judging, capacity, and mindfulness state (Table 1).

Significant negative correlations were observed between anxiety and the mindfulness capacities of describing, acting consciously, non-judging, and total mindfulness capacity, as well as between depression, stress, and the same set of mindfulness variables. Positive affect and self-esteem were positively correlated with mindfulness capacities and mindfulness state (Table 2).

**Table 2.** Correlational Values Between Variables at the Post-Intervention for Mindfulness Group.

Psychological measures	Mindfulness state and capacity						
	Observe	Describe	Act-conscious	Non-judge	Non- react	Mindfulness capacity	Mindfulness state
Anxiety	0.23	−0.38*	−0.55**	−0.44**	0.15	−0.41**	−0.02
Depression	0.06	−0.51**	−0.06**	−0.49**	−0.03	−0.62**	−0.17
Stress	0.15	−0.31*	−0.56**	−0.52**	0.27	−0.38*	−0.03
Positive	0.38*	0.29	0.19	−0.06	0.16	0.31*	0.515*
Negative	−0.21	0.04	0.19	0.22	−0.01	0.14	−0.16
Self-esteem	−0.02	0.29	0.25	0.29	0.03	0.35*	0.07

*Note. n* = 22.

* Significant correlation.

** Very significant correlation.

According to the qualitative analysis results, most of the mindfulness program participants considered that the practice of mindfulness exercises had a positive influence on their daily lives in the prison (86%) and that it could potentially influence their daily lives after release (90%). The main reasons mentioned (33%) were related to coping with anxiety and management of well-being, increased awareness (19%; e.g., “I really enjoyed the training, I learned some relaxation techniques that will help me calm down in more complicated situations” and enjoying practice (14%; e.g., “I consider it a good experience (. . .).” On a scale from 0 to 10 (corresponding to disliking the exercises being practiced to liking them very much), 95.2% of the participants indicated a value ≥5 (*M* = 7.92, *SD* = 2.45), 86% of them considered the program useful, and the remaining 14% did not find it useful. The main reason for perceived usefulness of the program was in helping control anxiety or to relax (43%; e.g., “. . . the way I started managing my time, relaxing on a day-to-day. . .”). Learning and practice new ways to solve problems was also mentioned by other participants (10%) as a benefit (e.g., “This program has taught me to be calmer, in desperate situations I try to solve it in the best way, calm, quiet (. . .) the exercises have led me . . . to know how to act more assertively”). An improved awareness of the body was also deemed useful (10%; e.g., Paying more attention to my body and the things around me, thus being able to enjoy life more, observing things and people with different eyes by pondering my actions and acting differently”), as well as the impact on well-being at the physical and mental levels (10%; e.g., “. . . it helped when my mind was wandering on less positive thoughts to draw my attention to myself through breathing leading to calming . . . physically and mentally”).

## Discussion

The purpose of this study was to verify the effectiveness of an MBI program in decreasing depressive symptoms, anxiety, stress, negative affect, and increasing positive affect, self-esteem, and mindfulness and ability in prisoners. Based on the results of this study, it was observed that the mindfulness training group showed an increase in self-esteem and mindfulness capacities. The qualitative analysis showed the usefulness of training for prisoners, not only in their daily prison life, but also post-release and the importance of focusing on breathing in a stressful and anxious situation.

The initial analysis was directed at comparing the two groups on the pre-test assessment. The two groups of inmates under analysis were relatively homogeneous in terms of their sociodemographic characteristics, except for literacy level. This difference between groups is related to the conditions imposed by the prison management team. The desire for randomization of participants into groups was not accomplished as only a rough approximated randomized scheme was possible. This criterion was imposed by the limitations of inmate/prison schedules and internal rules of security, and thus introduced a bias in the results. In addition, baseline results revealed significant differences between groups in a few psychological measures—perceived anxiety, perceived negative affect, perceived self-esteem, and perceived non-reacting mindfulness capacity. As such, the groups could not be considered similar in this aspect.

It is important to note that high levels of anxiety are characteristic in prison contexts; therefore, upon the pre-intervention assessment, the participants were exposed to a new situation—an extensive protocol that required reading and understanding the meaning of the sentences, which was thus an additional stress factor. The anxiety scale aimed to measure perceived feelings regarding the past week. However, the participants’ stress and anxiety may have most probably been dominated by their perceived feelings at the moment, and the level of difficulty of the current situation could explain the level of perceived anxiety being higher in the control group than in the mindfulness group at baseline.

Although the main prisoner condition stress factors were supposed to be the same for both groups, other factors besides the differences recorded could be explained by the characteristic emotional instability of individuals living in a prison. When looking at the differences found between the two groups at the baseline, a clear common pattern for all sets of variables was not defined, and the results were not always “better” in one group (the perceived negative affect was higher for the mindfulness group, the perceived self-esteem was higher for the mindfulness group, and the perceived non-reacting mindfulness capacity was higher for the control group). This suggests that psychological differences and emotional instability may also be the main explanatory factors for the differences found between groups at the baseline. The prison environment elicits negative emotions such as anger, fear, and helplessness, and is also associated with a lack of the ability to deal with them (Jang, 2020).

In the current study, no significant differences between groups were found at post-intervention for stress, anxiety, and depression. The mindfulness group’s average values were lower than those of the control group, but these differences were not significant, thus indicating that the hypothesis of the reduction of depressive symptoms, anxiety, and stress as an outcome of the intervention could not be confirmed, as previous study on MBIs have done (Weber et al., 2017).

In the present study, no significant differences were detected between the two groups in positive or negative affects post-intervention. These results and the lack of detection of changes in the mindfulness group between pre-and post-intervention concerning anxiety, stress, and depression should be associated with the maintenance of the affective states by individuals.

The lack of regular practice by the participants of the program, along with the fact that the meditative practice was limited to the mindfulness sessions contributed to this apparent lack of efficacy of the intervention concerning stress, anxiety, depression, and negative and positive affect. The results of different studies indicate that enactment, which entails building mindfulness practice into one’s daily life, is critical to continued outcomes. Daily practice in MBIs is very important (Bowen et al., 2014). However, the participants showed difficulties in daily practice in the prison environment.

Post-intervention results showed that the self-esteem of the mindfulness group was significantly higher than that of the control group. It should be noted that the change in self-esteem was positive for the mindfulness group and negative for the control group, thus suggesting that this was an effect of the program. Besides this associated uncertainty, it could be considered that the results confirm the initial hypothesis that a mindfulness intervention should improve inmates’ self-esteem. This result aligns with the findings of Samuelson et al. (2007) and Morley and Fulton (2020) who observed an increase in self-esteem in prisoners after a MBI.

The objective of MBIs is to increase awareness, and mindfulness practice has been associated with greater attentional capacity and greater body awareness (Greucci et al., 2015). In the present study, no significant differences were found between groups at post-intervention in any of the mindfulness measurements under consideration. Although the average values were almost always higher for the mindfulness group, changes were also registered in the mindfulness group in all mindfulness-related variables, and were significant for describing, non-reacting, observing, and total mindfulness capacities. Thus, despite the absence of differences between groups at post-intervention, these results strongly suggest the positive effect of the intervention in the domains of consciousness associated with mindfulness, as expected.

The results showed no significant changes between pre- and post-intervention in the control group for any of the variables under analysis. However, significant changes were observed in mindfulness variables in the mindfulness group (increase in capacities to observe, describe, and non-react), suggesting an improvement in participants’ well-being and an apparent broader efficacy of the MBI. The inexistence of significant changes in the control group from baseline to post-intervention allows an improvement in the certainty about changes occurring in the mindfulness group, and on the acceptance of the hypotheses of this study.

The correlation results demonstrate the benefits gleaned by the mindfulness group. The significant correlations, namely the negative association between anxiety (depression and stress) and mindfulness skills (describing, acting with awareness, and not judging), corroborate the potential increase in the ability to cope with stress and anxiety. Positive affect and self-esteem also showed a significant (positive) association with mindfulness variables (highest values of self-esteem and positive affect were associated with greatest mindfulness capacities and states), suggesting another possible positive effect of the intervention program. These results corroborate Muotka and Lappalainen’s (2018) study, which demonstrates a negative association between acceptance without judgment and depressive symptoms, as well as with the facets observed and act with awareness.

The qualitative results also strongly indicate the benefits of mindfulness training for prisoners, corroborating and reinforcing the quantitative results. As was perceived and reported by the participants following the program, the practice helped them deal with difficulties related to their prison life, such as uncomfortable situations, anxiety, and stress. Mindfulness exercises also helped them relax and the participants recognized their usefulness for their well-being. In relaxing the body and mind, some found mental peace, and due to that, were able to let go of their problems or find a way to solve them. The practice of focusing on breathing to cope with stressful situations was found to be the most useful, although other possible gains should be considered as well, such as being able to pay more attention to the things and people around, to be able to enjoy life more, and to take time to ponder their actions. The acquisition of the competence to pay attention to breathing in difficult situations appears to be useful for them in the future, mainly for their life outside the prison walls. Finally, the members of the prison management team and some of the prison guards said that they found the program to have a positive impact on prisoners, reporting that they noticed differences in the participants (such as being more quiet, less aggressive, and less impulsive). The qualitative results are in line with other studies, for example, Sumter et al. (2009) concluded that prisoners acquired a greater ability to relax, an improvement in anger management skills, and perceived well-being.

The present study has some limitations. The small sample size may have made some of the significant findings biased and constrained more robust data analysis. The failure to include a general measure of participants’ mental health and addiction history is also considered a limitation. It was also desirable that the self-report measures should be accompanied by physiological measures. And conducting a long-term follow-up assessment could have contributed to understanding the usefulness of a mindfulness intervention to prevent recurrence.

Future studies should consider these aspect adequately and further. In addition, the MBI protocols available clearly need to be adapted to the prison context to better suit these populations in order to enhance the effectiveness of the intervention. MBIs have already been proven to be beneficial for inmates (Bouw et al., 2019). However, studies have not been conclusive about the efficacy of such programs for inmates.

To conclude, the results of this study, were not enough to confirm the effectiveness of MBIs in reducing stress, anxiety, and depression among inmates, although a significant change in mindfulness-related conscious state and tendency was observed in the mindfulness group, and participants acquired perceived skills to cope with stress and anxiety. In sum, and despite the limitations noted, this study may contribute toward increasing the availability of empirical knowledge about the beneficial effects of MBIs on inmates, emphasizing the potential regulation of stress, anxiety, and depressive symptoms, and the improvement of self-esteem and mindfulness-related capacities and state.

## Supplemental Material

sj-docx-1-ijo-10.1177_0306624X221106333 – Supplemental material for Effects of a Mindfulness-Based Intervention on Male Portuguese Prisoners Supplemental material, sj-docx-1-ijo-10.1177_0306624X221106333 for Effects of a Mindfulness-Based Intervention on Male Portuguese Prisoners by Cláudia Carmo, Vivien Iacob, Marta Brás and Jacinta Fernandes in International Journal of Offender Therapy and Comparative Criminology
