# Supplementary material for: Effects of a Mindfulness-Based Intervention on Male Portuguese Prisoners
Source: Int J Offender Ther Comp Criminol. 2022 Jun 20;68(6-7):677–92. doi: 10.1177/0306624X221106333 (PMC11041068; doi:10.1177/0306624X221106333)
Supplement: sj-docx-1-ijo-10.1177_0306624X221106333 – Supplemental material for Effects of a Mindfulness-Based Intervention on Male Portuguese Prisoners [file sj-docx-1-ijo-10.1177_0306624X221106333.docx]

MANUSCRIPT ID: IJOTCC-21-0165

MANUSCRIPT TITLE: Effects of a Mindfulness-Based Intervention on Male Portuguese Prisoners

**Supplementary materials:**

Short description of the sessions:

a) Sessions 1 to 3: practice of yoga, body scan, awareness of breath, awareness of smell and taste; concept of mindfulness and mindfulness practice;

b) Sessions 4 to 6: exercises – practice of yoga, body scan, awareness of breath, awareness of sounds; the “monkey mind” and the self-regulation of attention on the present moment; dealing with thoughts and the difficulties of the practice;

c) Sessions 7 to 10: exercises – yoga, awareness of breath, awareness of sounds, walking meditation; focus on observation and description skills; writing/drawing exercise – recognizing emotions in the body; the stress response and the automatic pilot;

d) Sessions 11 to 14: practice of yoga and sitting meditation; “One-Minute Breathing Space" exercise; focus on thoughts and emotions; acceptance and non-judgment; writing exercise – behaviors, physical feelings, thoughts and emotions related to specific events;

e) Sessions 15 to 18: practice of yoga, mountain meditation and other sitting meditations; focus on acceptance and non-judgment; the meta-cognition; exploring coping with difficulties, stress and anxiety; mindfulness in a prisoner´ daily life. In a short synthesis, after the first sessions, mindful yoga clearly becomes the exercise that most attracted participants and promote them to calm down after arriving. keeping their interest and allowing them to be “present” in the session, mindful yoga had allowed the introduction and practice of the other mindfulness exercises. Instructions on the exercises, feedbacks and psychoeducation were always provided carefully considering the current condition of the individuals (incarcerated). For the same reason, issues such as non-judgmental attitude, kindness, compassion, and self-compassion, were shortly approached in a direct way.

The participants did not receive week's support materials due to security rules (all type of recorded guided meditation were not allowed to be provided and printed materials not recommended). Formal practice sheets and a few other written exercises have been performed by participants during some of the sessions.
